# *Ulmus parvifolia* Jacq. Exhibits Antiobesity Properties and Potentially Induces Browning of White Adipose Tissue

**DOI:** 10.1155/2020/9358563

**Published:** 2020-12-23

**Authors:** Yuan Yee Lee, Minki Kim, Muhammad Irfan, Heung Joo Yuk, Dong-Seon Kim, Seung Eun Lee, Seung-Hyung Kim, Suk Kim, Sung-Dae Kim, Man Hee Rhee

**Affiliations:** ^1^Laboratory of Physiology and Cell Signaling, College of Veterinary Medicine, Kyungpook National University, Daegu 41566, Republic of Korea; ^2^Herbal Medicine Research Division, Korea Institute of Oriental Medicine, Daejeon 34054, Republic of Korea; ^3^Department of Herbal Crop Research, National Institute of Horticultural and Herbal Science, Chungbuk 27709, Republic of Korea; ^4^Institute of Traditional Medicine and Bioscience, Daejeon University, Daejeon, Republic of Korea; ^5^Department of Veterinary Medicine, College of Veterinary Medicine, Gyeongsang National University, Jinju 52828, Republic of Korea; ^6^Research Center, Dongnam Institute of Radiological and Medical Sciences, Busan 46033, Republic of Korea

## Abstract

The bark of *Ulmus parvifolia* Jacq. (UP) was traditionally used as a diuretic and to treat intestinal inflammation. With modern evidence of the correlation of diuretics, gut inflammation, and obesity, our study has shown the antiobesity effects of the bark of UP. UP treatment reduced lipid production and adipogenic genes *in vitro*. *In vivo* studies revealed that UP 100 mg/kg and UP 300 mg/kg treatment significantly reduced mouse weight without reducing food intake, indicating increased energy expenditure. UP significantly reduced the weight of epididymal and subcutaneous adipose tissue and decreased liver weight. Histological analysis revealed improvement in the progression of nonalcoholic fatty liver disease and epididymal white adipose tissue hypertrophy induced by a HFD. Real-Time PCR of epididymal adipose tissue revealed significant increases of uncoupling protein-1 (UCP-1) and peroxisome proliferator-activated receptor gamma coactivator 1-alpha (PGC-1*α*) expression after UP 300 mg/kg treatments. Phosphorylation of AMP-activated protein *α* (AMPK*α*) was increased, while phosphorylation of Acetyl-CoA Carboxylase (ACC) was reduced. Our findings reveal the ability of UP to reduce the occurrence of obesity through increased browning of white adipose tissue via increased AMPK*α*, PPAR*γ*, PGC-1*α*, and UCP-1 expression.

## 1. Introduction

The *Ulmus* genus are elm trees found in North America [1], the Himalayas, and East Asia [2]. *Ulmus parvifolia* Jacq. (UP) which is native to Japan, Korea, and China has been reported for its antioxidant and anti-inflammatory activities [3]. The leaves of UP were traditionally used as an external dressing on wounds and ulcerous tissue [4] and as a lithontripic agent [5]. According to the Chinese Supplement to Materia Medica (Bencao Gangmu Shiyi), the bark of UP is nontoxic and was used to treat strangury, burns, and intestinal inflammation [6]. Its bark was also used for its demulcent and lithontripic properties other than treating cough and fever. It was also used as a diuretic [7]. Insulin resistance was said to be related to hypertension as the release of free fatty acids due to excess adipose tissue lipolysis induces various metabolic abnormalities, as well as vascular dysfunction. There was also evidence suggesting that peptides derived from adipocytes may affect arterial pressure, contributing to hypertension [8,9]. Diuretics have also been commonly used to treat obesity [10,11]. In obesity, adipocytes secrete proinflammatory cytokines (TNF-*α* and IL-6), also known as adipokines, into the circulation [12]. The ability of UP to treat burn injury and intestinal inflammation suggests its anti-inflammatory properties that could inhibit the proinflammatory state in adipose tissue. Gut anti-inflammatory agents were also used to regulate obesity-related insulin resistance [13], which suggests that UP may also be used to treat obesity as UP was traditionally used to treat intestinal inflammation. We have also reported that UP exhibits antiplatelet and antithrombotic activity [14]. Existing traditional and modern evidence and reports suggest that UP may exhibit antiobesity properties.

UP was also reported to inhibit nitric oxide production in lipopolysaccharide-treated RAW 264.7 murine macrophages [15], exhibit anticancer and antiviral properties [16], and accelerates skin wound healing [17]. However, there were no reports on the antiobesity effects of the bark extract of UP. Fat tissue is an organ that serves as a survival adaptation in humans by providing a source of energy during starvation and heat insulation in cold weather. Adipose tissue exists in two forms, white adipose tissue (WAT) and brown adipose tissue (BAT) [18]. Triglycerides are stored in WAT as lipids in unilocular white adipocytes. Excessive accumulation of WAT has been shown to lead to cardiovascular diseases [19], type 2 diabetes [20], and cancer [21]. BAT dissipates heat due to the activity of uncoupling protein-1 (UCP-1). Hence, BAT is active metabolically. UCP-1 functions through the dissipation of the proton gradient into the inner mitochondrial membrane [22]. Increasing UCP-1 expression increases the conversion of free fatty acids during heat dissipation, hence reducing the amount of triglycerides in the body. This could potentially be one target for therapeutic methods for reducing the incidence of obesity.

In this study, we investigate the antiobesity effects of ethanol extracts of the bark of UP *in vitro* using 3T3-L1 preadipocytes and *in vivo* in a model of mice fed a high-fat diet (HFD). Our study demonstrates that UP inhibits lipid generation and adipocyte differentiation and also inhibits expression of various adipogenetic genes. *In vivo* studies reveal effective inhibition of HFD-induced obesity, as well as decreased serum triglyceride levels, decreased body weight, and reduction of fatty liver. Orlistat, also known as tetrahydrolipstatin, is an inhibitor of gastric and pancreatic lipases. It acts in the gastrointestinal (GI) lumen, and it is also a widely used pharmacological drug to treat obesity [23]. Although orlistat has produced positive results in randomized placebo-controlled trials [24], there have been occurrences of fatty and oily stool and faecal urgency in patients [25]. Hence, our study aims to discover natural health supplement alternatives with the potential to treat obesity.

Our study has also revealed the ability of UP to increase metabolic activity via increased the expression of *UCP-1* and *PGC-1α* in WAT. UP has increased browning of WAT. Therefore, energy expenditure is increased by increasing thermogenesis. However, more studies should be conducted to confirm the conversion of triglycerides through fatty acid oxidation and the role of Sirtuin 1 (Sirt1) in mitochondrial biogenesis in conjunction with treatment with UP.

## 2. Materials and Methods

### 2.1. Reagents

The bark of UP was kindly provided by the Herbal Crop Research department of the Korean National Institute of Horticultural and Herbal Science (voucher number: NIHHS-0169), which is sourced from Danyang Province, Chungbuk, Republic of Korea. UP was verified by Professor Lee Seung Eun. Dulbecco's modified Eagle Medium (DMEM), fetal bovine serum (FBS), streptomycin, and penicillin were purchased from Welgene (Daegu, Republic of Korea). Insulin, 3-isobutyl-1-methylxanthine (IBMX), indomethacin, Oil Red O, orlistat, and neutral buffered formalin were purchased from Sigma-Aldrich (St. Louis, MI, USA). TRIzol reagent was purchased from Invitrogen (Carlsbad, CA, USA). Primers used in this study displayed in Table 1 were purchased from Bioneer (Daejeon, South Korea). Antibodies for p-AMPK*α*, AMPK*α*, p-ACC, and *β*-actin were purchased from Cell Signaling Technology (Danvers, MA, USA). Standards of (+)-catechin (product no. ES090-A) and catechin-7-*O*-*β*-D-apiofuranoside (product no. ES060-A) were purchased from Ensol Biosciences Inc. (Daejeon, Republic of Korea), both having a purity ≥95%.

### 2.2. Preparation of *U. parvifolia*

Bark of UP was collected and shredded. The bark was extracted using 70% ethanol at 80°C, filtered through filter paper (Whatman, USA), and condensed using a rotary evaporator before lyophilization to obtain the powder form of the extracts and weighed according to the desired concentrations.

### 2.3. UPLC-QTof MS Analysis of *U. parvifolia*

A UPLC system (Waters Corp., Milford, MA, USA) equipped with a binary solvent delivery system, an autosampler, and a UV detector was used. Briefly, aliquots of 2.0 *μ*L of UP were injected into a BEH C_18_ column (2.1 × 100 mm × 1.7 *μ*m) at a flow rate of 0.4 mL/min and were eluted with a chromatographic gradient consisting of two mobile phases, which are A, water containing 0.1% formic acid; B, acetonitrile containing 0.1% formic acid. A linear gradient was optimized: 0 min, 5%; 0–8 min, 5–15% B; 8–11 min, 15–80% B; 11-12 min, 80–100% B; 12–13.3 min, 100% B; and 13.4–15 min, back to 5% B. Using a negative ion mode with a capillary voltage of 2.3 kV, cone voltage of 50 V, source temperature of 110°C, and a desolvation temperature of 350°C, the quadrupole time-of-flight mass spectrometer (Q-Tof Premier™, Waters Corp., Milford, MA, USA) was operated. A reference solution of leucine-enkephalin ([M − H]^−^*m*/*z* 554.2615) in the form of a spray was used as the lock mass. The full-scan data and the MS/MS spectra were collected with MassLynx software (Thermo Fisher, MA, USA).

### 2.4. GCMS Analysis of *U. parvifolia*

An Agilent 7890A GC (Agilent Technologies, Santa Clara, CA, USA) with a 30 m × 0.25 mm i.d. DB-5MS column and an Agilent 5975C mass selective detector (MSD) were used to separate and quantify the constituents of UP. Samples were injected in split mode with a temperature of 250°C. The transfer line temperature was 280°C, and the ion source temperature was 230°C. The column temperature was held at an initial temperature of 70°C for 1 min and was raised to 300°C at a rate of 5°C/min and held at a final temperature of 300°C for 30 min. Helium was used as a carrier gas at a constant flow rate of 1 mL/min. Mass spectrometry was performed using the electron ionization (EI) and scan modes.

### 2.5. Cell Culture

3T3-L1 preadipocytes purchased from ATCC (Manassas, VA, USA) were cultured in Dulbecco's Modified Eagle Medium (DMEM) supplemented with 10% fetal bovine serum, 100 IU/mL penicillin, and streptomycin and maintained at 5% CO_2_ and 37°C. Differentiation was induced with media supplemented with insulin, indomethacin, and IBMX. Cells were allowed to differentiate for 3 days and then cultured with normal media supplemented with insulin for postdifferentiation maintenance. Cells were differentiated for a total of 10 days for consecutive experiments.

### 2.6. Oil Red O Staining

Cells were differentiated using differentiation media and simultaneously treated with 6.25, 12.5, and 25 *μ*g/mL of UP. After 10 days, the cells were stained with Oil Red O staining. Oil Red O solution was then added to plates cultured with 3T3-L1 cells for visualization of lipids secreted by the cells under a microscope.

### 2.7. Cell Viability Assay

Viability of undifferentiated 3T3- L1 cells were assessed by using a 3-(4,5-dimethylthiazol-2-yl)-2,5-diphenyltetrazolium bromide (MTT) assay. The cells were seeded in 24-well plates for 24 h. Incubation of UP in specified concentrations was performed. Proceeding steps were carried out as previously reported [26]. Briefly, MTT was added to wells and left to incubate for 3 h. DMSO was added to each well to dissolve violet crystals of MTT and left on a rocker for 10 min. Plates were then read at 560 nm using a plate reader.

### 2.8. High-Fat Diet Induced Obesity in Mice

All experiments were approved by the Institutional Animal Care Committee of Kyungpook National University in accordance to NIH guidelines (approval number: 2018-0117). Four-week-old male ICR mice were purchased from Orient Bio (Gyeonggi-do, Republic of Korea), maintained in a 12-h light/dark controlled room with regulated temperature at 22 ± 2°C and humidity of 50 ± 10%. The mice were given access to chow and water *ad libitum* and allowed to acclimatize for one week, grouped in numbers of 6 with a total of 5 groups. Six mice were given normal chow, and the remaining mice were fed an HFD (D12492; Research Diets, New Brunswick, NJ, USA). Mouse weight, food intake, and water intake were measured weekly. After four weeks of a HFD, mice were given an oral administration of orlistat (10 mg/kg) and UP at 100 mg/kg and 300 mg/kg for an additional 8 weeks. Twenty-four hours after the final administration, mice were anaesthetized and blood was collected by cardiac puncture. Organs and adipose tissue were immediately harvested, weighed, frozen, and fixed in neutral buffered formalin. The food efficiency ratio (FER) was calculated as the amount of intake per mouse divided by the weight of the mouse.

### 2.9. RT-PCR and Real-Time PCR of 3T3-L1 Preadipocytes and Epididymal Adipose Tissue of Mice

RNA was extracted from 3T3-L1 preadipocytes and epididymal adipose tissue from HFD mice using TRIzol solution. Brown adipose tissue was extracted from mice fed with normal chow and used as a positive control for browning genes. Proceeding steps were conducted as reported [27]. Briefly, RNA was separated using chloroform and purified using alcohol. The RNA was resuspended in DEPC-DW, and the concentration was measured using a nanophotometer. Reverse-transcriptase PCR was conducted using a premix (Bioneer, Daejeon, Republic of Korea). Real-Time PCR was then conducted with the resultant cDNA using target primers of adipogenesis-related genes and browning-related genes, as shown in Table 1. For *C/EBPα* and *PPARγ*, RNA was extracted at day 5 of differentiation, while RNA was extracted at day 8 for other target genes.

### 2.10. Western Blot Analysis of Epididymal Adipose Tissue and Liver Tissue

Proteins were extracted from 3T3-L1 cells and liver tissues of mice and analyzed using western blot analysis. Cells and liver tissues were homogenized with Pro-Prep protein lysis solution (Invitrogen, Daejeon, Republic of Korea), and protein concentrations were analyzed using the Bradford method. Proceeding procedures were conducted as previously reported [28]. Briefly, proteins were separated using 10% SDS-PAGE and transferred to a PVDF membrane, followed by blocking with 5% skim milk at room temperature for 1 h. The membranes were then incubated with the respective primary antibodies (1 : 3,000) overnight, followed by incubation with a secondary antibody (1 : 1,000) at room temperature for 90 min. The membranes were then washed with 1% Tween-20 TBS before developing the membranes using ECL chemiluminescence in a gel developer (General Electric, Boston, MA, USA). Western blot analysis was repeated in triplicate, and the relative expressions were quantified using ImageJ software (NIH, USA).

### 2.11. Serum Chemistry

Collected blood was allowed to separate for 2 h and centrifuged at 3,000 rpm for 15 min. Serum was collected and analyzed using a blood analyzer for triglyceride, glucose, total cholesterol, LDL, HDL, ALT, and AST levels.

### 2.12. Hematoxylin and Eosin (H&E) Staining

Harvested liver tissue and epididymal adipose tissue were directly fixed in neutral buffered formalin after harvesting and weighing. Images of the slides were acquired using a Nikon Eclipse E6000 microscope (Nikon, Minato-ku, Tokyo, Japan). Dehydrated tissues were fixed in paraffin and sectioned before staining with H&E. Size of adipocytes was determined using AdipoCount [29].

### 2.13. Statistical Analysis

Statistical significance was analyzed using Graphpad Prism version 7.00 (San Diego, CA, USA) and one-way ANOVA with Dunnett's posttest. *P* < 0.05 was considered significant. Data were presented as mean ± SD.

## 3. Results

### 3.1. UPLC-QTof MS Analysis and GCMS Analysis of *U. parvifolia*

**Figure 1:**
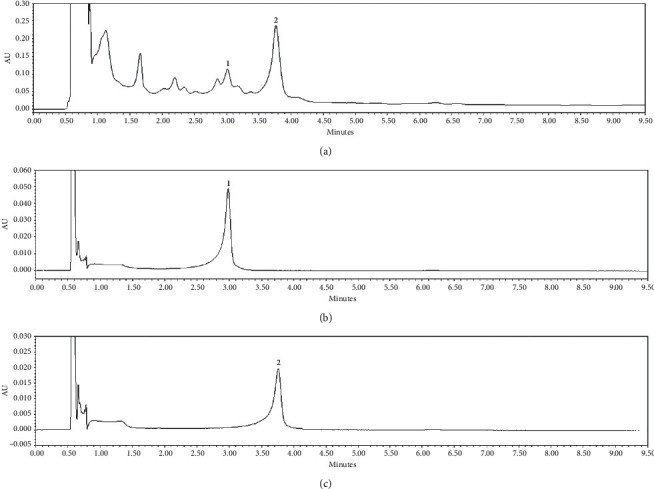
*UPLC-QTof MS* identification of UP. Peaks detected in ethanol extract of the bark of UP (a). Peaks 1 and 2 were quantified by using a UV detector at 280 nm for target catechin glycosides. Peak 1 was identified as +(-) catechin as compared to its standard shown in (b), and peak 2 was identified as catechin-7-O-*β*-D-apiofuranoside as compared to its standard as shown in (c).

Using *UPLC-QTof MS*, the compounds identified in ethanol extract of the bark of UP are as shown in Figure 1(a). Peak 1 was identified as +(-) catechin as compared to its standard shown in Figure 1(b), and peak 2 was identified as catechin-7-O-*β*-D-apiofuranoside as compared to its standard, as shown in Figure 1(c). The concentration of catechin detected is 6.14 mg/g, whereas that of catechin-7-O-*β*-D-apiofuranoside was 156.3 mg/g, as previously described [14]. GCMS analysis revealed that the main components are hexadecanoic acid and *β*-sitosterol (Table 2).

### 3.2. *U. parvifolia* Inhibited Production of Lipid in 3T3-L1 Preadipocytes

**Figure 2:**
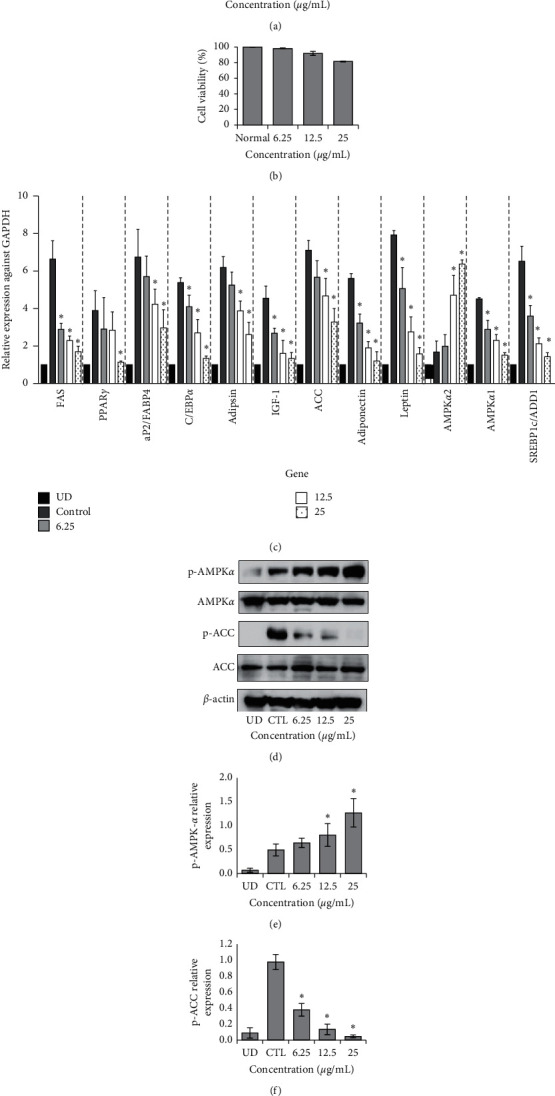
UP inhibits secretion of lipid droplets in 3T3-L1 preadipocytes. 3T3-L1 preadipocytes were differentiated or undifferentiated (UD) and treated with or without UP for 10 days before they were stained with oil red O (a). Cell viability was confirmed with an MTT assay after 3T3-L1 cells were treated with different concentrations of UP (b). Real-Time PCR was carried out by extracting RNA of differentiated 3T3-L1 cells with or without treatment of UP (c). Western blot analysis of p-AMPK*α* and p-ACC against the housekeeping gene *β*-Actin. 3T3-L1 cells were treated with or without UP after differentiation. After 10 days, protein was extracted from the cells, separated using SDS-PAGE, transferred to a PVDF membrane, incubated overnight with the primary antibody, incubated with secondary antibody, and developed (d). Expressions of genes were compared against the differentiated control group. Western blot was repeated in triplicate, and images were quantified using ImageJ (e and f). Statistical analysis was performed using one-way ANOVA with Dunnett's posttest, and *P* < 0.05 was considered significant.

Lipid is produced when preadipocytes differentiate into adipocytes. UP reduced lipid production in differentiated 3T3-L1 preadipocytes (Figure 2(a)). The concentrations of UP used were not toxic, as determined by an MTT assay on 3T3-L1 cells (Figure 2(b)). Expression of adipogenic genes *FAS*, *PPARγ*, *aP2/FABP4*, *C/EBPα*, *adipsin*, *IGF-1*, *ACC*, *adiponectin*, *leptin*, *AMPKα1*, and *SREBP1c/ADD1* was decreased with increasing concentrations of UP treatment (Figure 2(c)). However, *AMPKα2* expression was increased. AMPK is a main regulator of metabolism, and its phosphorylated form was increased with increasing concentrations of UP treatment, while p-ACC protein expression was reduced (Figure 2(d)). No change was observed in ACC expression. The relative expressions of AMPK*α* and ACC were quantified using ImageJ software (NIH, USA), as shown in Figures 2(e) and 2(f).

### 3.3. *U. parvifolia* Inhibits Development of Obesity in Mice Fed an HFD

**Figure 3:**
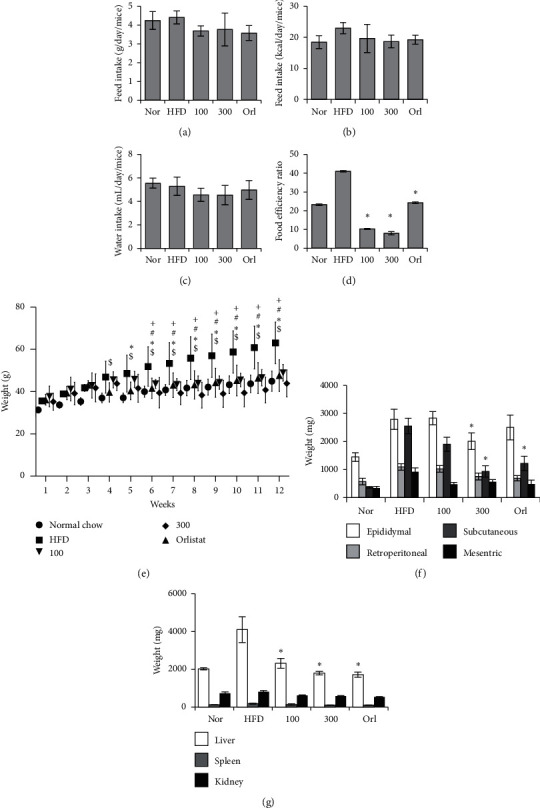
UP reduced obesity in mice. Food intake and water intake were monitored weekly and averaged as shown in (a–c). Food efficiency ratio was calculated using the weight gain to intake of food ratio (d). Weight of mice for each group was recorded every week for a period of 12 weeks. Mice in the normal group (nor) were given normal chow, whereas all other groups were given a HFD. After 4 weeks, oral administration of orlistat and UP was given daily for 8 weeks (e). After 12 weeks, mice were euthanized, and the fat tissue, liver, spleen, and kidney were harvested and weighed immediately (f and g). Statistics were analyzed using one-way ANOVA with Dunnett's posttest, and *P* < 0.05 was considered significant. Statistical significance of HFD as compared to the control group in (d) are indicated by $; ^*∗*^, for the orlistat group against HFD; #, for UP 100 against HFD; and +, for UP 300 against HFD.

There were no significant differences in food and water intake between the groups (Figures 3(a)–3(c)). However, mice that were administered orlistat daily and UP 100 or UP 300 exhibited notable decreases in weight (Figure 3(e)). Orlistat group mice exhibited a significant reduction as compared to mice consuming only an HFD from week 5, whereas UP 100- and 300-treated groups exhibited a significant reduction from week 6. FER was significantly reduced in both UP-treated groups in a dose-dependent manner (Figure 3(d)). Subcutaneous adipose tissues were significantly reduced in the orlistat and UP 300-treated groups, whereas epididymal adipose tissue was reduced significantly only in UP 300 (Figure 3(f)). The weight of the liver has decreased in treated groups, whereas there was no significant change in kidney and spleen weight in all groups (Figure 3(g)). From the results, it can be seen that UP treatment significantly reversed weight gain in mice and reduced FER and weight of adipose tissue and the liver.

### 3.4. *U. parvifolia* Improved Histological Damage Induced by an HFD in Mice

**Figure 4:**
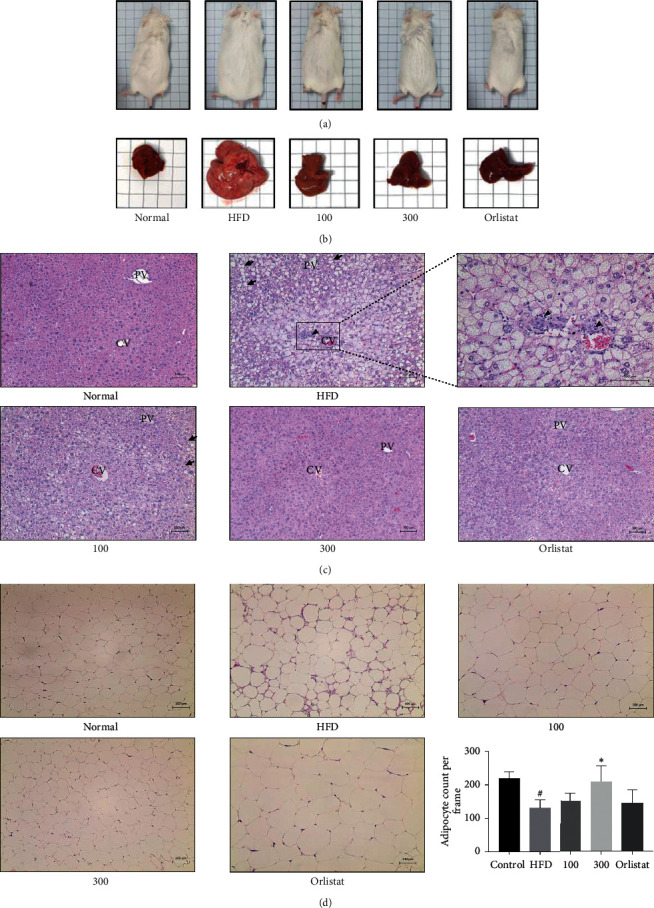
UP reduced the size of mice and mouse livers and improved the histology of the liver and adipose tissue induced by an HFD. Mice were fed with an HFD for a total of 12 weeks, and oral administration of orlistat and UP was carried out after 4 weeks of a daily HFD. Representative images of mice and livers for each group (a-b). Liver tissues and epididymal adipose tissue were dehydrated and fixed in paraffin and then sectioned before staining with H&E (c-d). Liver tissues (c) and epididymal adipose tissue (d) were observed at 100x. Arrows indicate balloon cells, and arrowheads indicate the infiltration of inflammatory cells. CV indicates the location of the central vein, whereas PV indicates the portal veins. Magnified image of the CV area of the liver tissue in HFD-treated group was taken at 200x. Statistical analysis for adipocyte count performed using one-way ANOVA with Dunnett's posttest, and ^*∗*^ indicates *P* < 0.05 compared to the HFD group, whereas ^#^ indicates a *P* < 0.05 as compared to the control group.

There was a visible change in body size as shown in the representative pictures of mice for each group; mice were significantly increased in size in the HFD-group, whereas there was a visible reduction in size in mice from the groups treated with orlistat and UP 100 or UP 300 (Figure 4(a)). Liver tissue extracted after the mice were euthanized was compared (Figure 4(b)). There was a visible increase in size and change in coloration to a paler shade, indicating the occurrence of fatty liver after consuming an HFD. This change was improved with orlistat and UP 100 or UP 300 treatment. This observation was further confirmed with histological analysis of liver tissue and epididymal adipose tissue stained with H&E. The hepatocytes in HFD-treated mice were remarkably increased in size, and the pale coloration was visible due to accumulation of lipids. There was also a visible decrease in size of hepatocytes in the orlistat and UP-treated groups. Ballooning of hepatocytes, macrovesicular steatosis (indicated by arrows), and the foamy characteristics of the hepatocytes as depicted in microvesicular steatosis were also identified in the HFD-treated group, indicating the occurrence of nonalcoholic fatty liver disease (NAFLD). Infiltration of inflammatory cells was also observed (indicated by arrowheads) (Figure 4(c)). Therefore, treatment of orlistat and UP has remarkably inhibited the progression of NAFLD. The size of adipocytes in HFD-treated mice was significantly larger, as compared to mice fed normal chow, indicating hypertrophy of adipocytes. Adipocytes were shown to decrease with treatment of orlistat and UP. UP 300 in particular reduced the size of adipocytes as compared to the adipocytes of mice fed normal chow. Adipocyte count per frame was also significantly recovered in UP 300-treated mice (Figure 4(d)). This has confirmed the efficacy of UP in reversing obesity induced with an HFD and demonstrates the therapeutic potential of this compound for increasing metabolism and reversing obesity.

### 3.5. *U. parvifolia* Increased Browning-Related Genes in Adipose WAT

**Figure 5:**
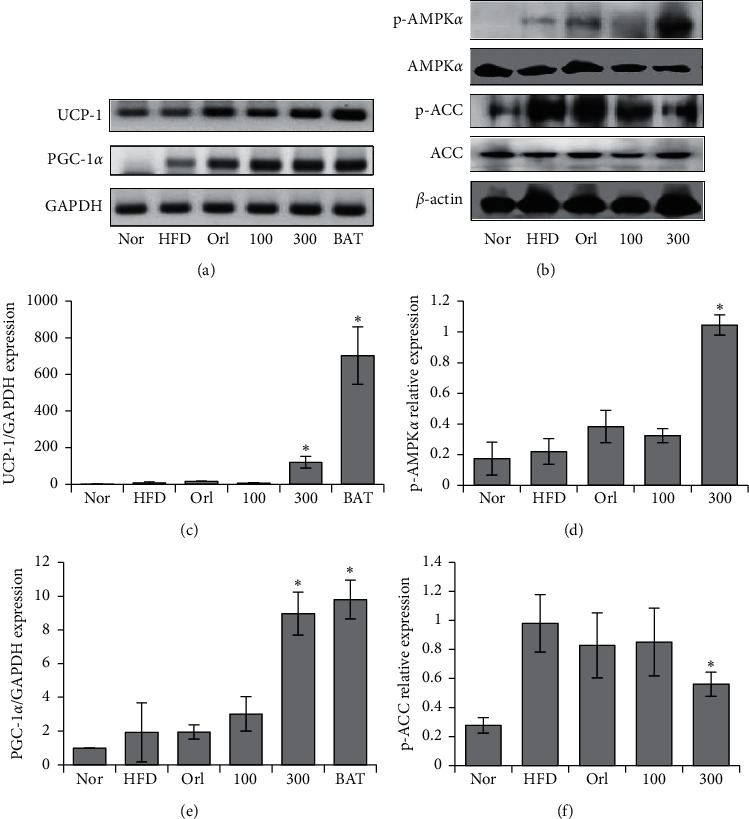
UP increased expression of browning markers in mice. Extracted RNA was reverse transcribed, and the browning related genes *UCP-1* and *PGC-1α* were investigated using Real-Time PCR conducted using the resultant cDNA (a). Quantification of gel images (c and e). Protein expressions of p-AMPK*α* and p-ACC in liver tissue were investigated using western blot analysis, as shown in (b), and the gel images were quantified using ImageJ (d and f). All experiments were conducted in triplicate. Statistics were analyzed using one-way ANOVA, and *P* < 0.05 was considered significant.

Using real-time PCR, the expression of *PGC-1α* and *UCP-1* were shown to increase in particular with treatment of UP 300 (Figures 5(c) and 5(e)). The products of real-time PCR were run on ethidium bromide-stained agarose gel for confirmation (Figure 5(a)). The protein expression of p-AMPK*α* was increased significantly, whereas the expression of p-ACC was decreased only in UP 300 (Figure 5(b)). No change was observed in ACC expression. The gel images were quantified using ImageJ (Figures 5(d) and 5(f)). Our results have shown that UP has induced browning of epididymal adipose tissue in mice fed an HFD.

### 3.6. Serum Biochemistry of Mice in a High-Fat Model

**Figure 6:**
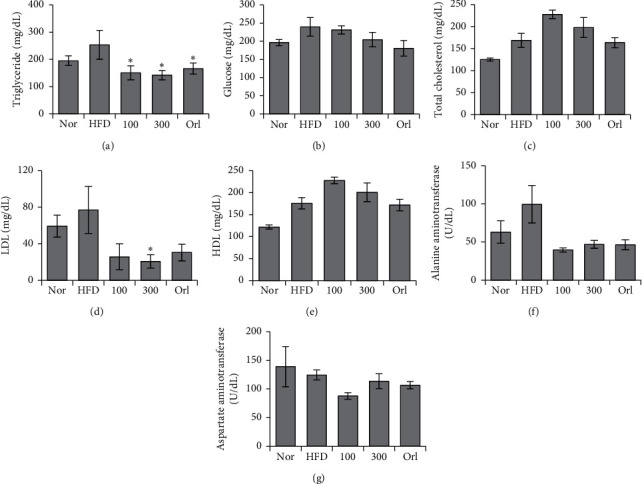
UP improved serum biochemical concentrations induced by an HFD in mice. Blood was collected by cardiac puncture, and serum was separated. Levels of (a) triglyceride, (b) glucose, (c) total cholesterol, (d) LDL, (e) HDL, (f) ALT, and (g) AST were investigated using a blood analyzer. Statistical analysis was performed using one-way ANOVA with Dunnett's posttest, and *P* < 0.05 was considered significant.

Serum triglyceride levels were significantly reduced by orlistat and UP 100 or UP 300 treatment. LDL levels were decreased with UP treatment. However, there were no significant decreases in glucose levels, total cholesterol, and HDL in any treated groups. ALT and AST are both markers of liver damage and did not show significant change in all groups (Figures 6(a)–6(g)).

## 4. Discussion

Other than storing lipids, white adipose tissue also functions as an endocrine organ, secreting mainly adiponectin and leptin [30]. Additionally, white adipose tissues secrete TNF-*α* in an obese rodent model [31]. IL-6 was also detected in white adipose tissues in cases of obesity and insulin resistance [32]. Both TNF-*α* and IL-6 are also known as adipokines and proinflammatory cytokines. Secretion of adipokines by WAT is a response to hypoxia in areas of fat deposits in obese individuals, as the vasculature is insufficient to maintain normoxia throughout the WAT due to its constant expansion. This causes the adipocytes to be hypoxic, and an inflammatory response increases blood flow and stimulates angiogenesis [33]. Therefore, obesity should be treated not only merely as a metabolic disorder but also as the root cause of many chronic diseases.

3T3-L1 preadipocytes were used to investigate adipogenesis *in vitro*. Peroxisome proliferator-activated receptor *γ* (*PPARγ*) and CCAAT/enhancer binding protein *α* (*C/EBPα*) are responsible for preadipocyte growth arrest and their differentiation into adipocytes. As the shift in gene expression indicates cellular differentiation, we studied the related gene expression for confirmation of preadipocyte differentiation. Our results have shown that *PPARγ*, *C/EBPα*, and *SREBP1c/ADD1* are downregulated in 3T3-L1 preadipocytes after treatment with UP. Furthermore, *leptin* and *adiponectin* are also adipokines secreted by adipose tissue. *Leptin* in serum is elevated in the case of obesity as it functions to accelerate energy expenditure [34], whereas insulin levels are negatively correlated with adiponectin levels in 3T3-L1 preadipocytes [35]. Treatment with UP also reduced both *leptin* and *adiponectin* mRNA levels in 3T3-L1 preadipocytes in this study. *Adipsin* is upregulated in the presence of insulin in 3T3-L1 preadipocytes [36]. As *FAS*, *ACC*, and *aP2/FABP4* are regulated by *PPARγ* [37,38], these genes were downregulated with UP treatment. *Insulin growth factor 1 (IGF-1)* is essential in the differentiation of 3T3-L1 preadipocytes [39], and its expression has been reduced with treatment of UP. AMPK is the master regulator of metabolism. Phosphorylation of AMPK inhibits the phosphorylation of ACC which then inhibits lipid synthesis and, at the same time, promotes fatty acid oxidation by increasing CPT1 (carnitine palmitoyltransferase 1) expression. As leptin inhibits the phosphorylation of *AMPKα2* [40], suppression of leptin explains the increase of *AMPKα2* (Figure 2(c)). UP has been shown to reduce lipid production and did not show signs of cytotoxicity in 3T3-L1 preadipocytes for the concentrations of UP used *in vitro* and additionally resulted in the downregulation of mRNA expressions of adipogenesis-related genes and increased expression of *AMPKα2*. Western blot analysis has also revealed that UP is capable of upregulating p-AMPK*α* and suppressing p-ACC (Figures 2(d)–2(f)).

*PGC-1α* was previously identified to be expressed only in BAT. Recent studies revealed the browning of WAT, also known as brite or inducible brown adipocytes [41]. Brown adipocytes are dominant in BAT, whereas beige adipocytes are found in WAT in cases of increased energy expenditure or exposure to cold. Therefore, the increase of beige adipocytes is marked by increases of *PGC-1α* and *UCP-1* and results in increased energy expenditure that can then counter obesity. Brown adipocytes have large numbers of mitochondria and highly express *UCP-1*, which is located in the inner mitochondrial membrane. Fatty acids from triglycerides will undergo *β*-oxidation followed by conversion into chemical energy by mitochondrial *UCP-1* [42]. Therefore, increasing the expression of *PGC-1α* and *UCP-1* in white adipose tissue increases energy expenditure, which will reduce the amount of triglycerides stored in WAT. Our results have shown that mice fed with UP have markedly increased *PGC-1α* and *UCP-1* expression in the epididymal adipose tissue of mice (Figure 5), indicating that UP increases the browning of WAT. The reduced weight of the mice and the respective adipose tissues in mice indicated the conversion of triglycerides in WAT to fatty acids which are then dissipated into heat energy through conversion by *UCP-1*, with no observed reduction of feed intake in all groups. UP also upregulated p-AMPK*α* expression and downregulated p-ACC expression in epididymal adipose tissue of mice fed a HFD, confirming the role of UP in countering obesity. These findings are further supported by evaluation of the levels of serum biomarkers, namely triglyceride, glucose, cholesterol, ALT, and AST, accompanied by the improvement in histological analysis of liver tissue and epididymal adipose tissue (Figures 4 and 6). UP increased lipid metabolism as there were lesser fat droplets deposited in the liver as observed in H&E staining, preventing NAFLD as previously reported [43].

*UPLC-QTof MS* was carried to identify the polar compounds in UP. Our findings revealed that the most abundant polar compounds in UP are catechin and catechin-7-*O*-*β*-D-apiofuranoside (Figure 1). Catechin has been know for antiobesity properties, explaining its possible contribution in the efficacy of UP in curbing obesity [44]. Catechin-7-*O*-*β*-D-apiofuranoside prevents hepatic fibrogenesis by inhibiting stellate cell activation [45]. GCMS analysis was carried out to identify the nonpolar constituents of UP. Our findings have shown that the major nonpolar compounds are hexadecanoic acid and *β*-sitosterol (Table 2). While hexadecanoic acid has been reported to have anti-inflammatory properties [46], *β*-sitosterol has been reported to induce apoptosis of cancer cells, have hypocholesterolemic and antidiabetic properties, and increase the activity of NK cells [47]. Moreover, sitosterol has also been reported to reduce choric inflammation induced by obesity [48]. Taken altogether, we believe that *β*-sitosterol and catechin in UP are the main ingredients that induce WAT browning.

## 5. Conclusions

**Figure 7:**
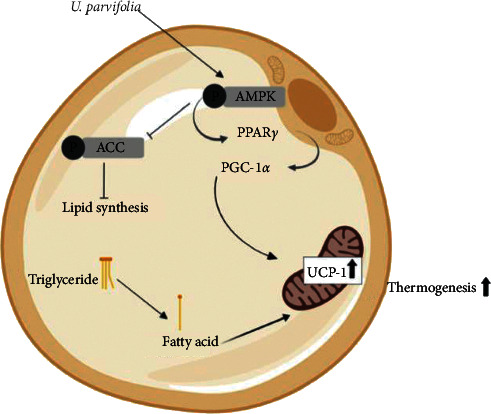
Schematic diagram of the proposed mechanism of action of UP. UP increased the browning of WAT by acting through increased AMPK*α* expression, which, in turn, increases *PPARγ* expression followed by the increase of *PGC-1α* expression, which induces upregulation of mitochondrial *UCP-1* in white adipocytes and increases the usage of stored triglycerides and thermogenesis. Phosphorylation of ACC was also inhibited, subsequently inhibiting lipid synthesis.

Our findings reveal that UP increased browning in WAT in mice, possibly acting through the PGC-1*α*/SIRT1/UCP-1 axis. Further studies should be conducted to confirm the role of SIRT1 and PRDM16. In conclusion, we have revealed the mechanism of action of UP on white adipocytes is via increased mitochondrial biogenesis in WAT, inducing an increase in energy expenditure and thermogenesis (Figure 7).

## Figures and Tables

**Table 1:** Primer sequences for real-time PCR.

Gene	Primer	Sequence
FAS	Forward	5′–CTGAGATCCCAGCACTTCTTGA–3′
	Reverse	5′–GCCTCCGAAGCCAAATGAG–3′
PPAR*γ*	FAM	5′–TCGGAATCAGCTCTGTGGACCTCTCC–3′
aP2/FABP4	Forward	5′–TGGGAACCTGGAAGCTTGTCTC–3′
	Reverse	5′–GAATTCCACGCCCAGTTTGA–3′
C/EBP*α*	Forward	5′–TGGACAAGAACAGCAACGAGTAC–3′
	Reverse	5′–CGGTCATTGTCACTGGTCAACT–3′
Adipsin	Forward	5′–CACCATCGACCACGACCTC–3′
	Reverse	5′–AGTGTGGCCTTCTCCGACAG–3′
IGF-1	Forward	5′–CCGTCGATAGTGGCATCCATGAAAC–3′
	Reverse	5′–GGACCAATACCTGCTATAGGG–3′
ACC	Forward	5′–ATTGTGGCTCAAACTGCAGGT–3′
	Reverse	5′–GCCAATCCACTCGAAGACCA–3′
Adiponectin	Forward	5′–GTCTCAGCTGTCGGTCTTCCCCT–3′
	Reverse	5′–CCCTGGCTTTATGCTCTTTGC–3′
Leptin	Forward	5′–CCAAAACCCTCATCAAGACC–3′
	Reverse	5′–GTCCAACTGTTGAAGAATGTCCC–3′
AMPK*α*1	Forward	5′–AAGCCGACCCAATGACATCA–3′
	Reverse	5′–CTTCCTTCGTACACGCAAAT–3′
AMPK*α*2	Forward	5′–GATGATGAGGTGGTGGA–3′
	Reverse	5′–GCCGAGGACAAAGTGC–3′
SREBP1c/ADD1	Forward	5′–AGCCTGGCCATCTGTGAGAA–3′
	Reverse	5′–CAGACTGGTACGGGCCACAA–3′
PGC-1*α*	Forward	5′–AAGACAGGTGCCTTCAGTTCACTCTCAG–3′
	Reverse	5′–AGCAGCACACTCTATGTCACTCCATACAG–3′
UCP-1	Forward	5′–ACTGCCACACCTCCAGTCATT–3′
	Reverse	5′–CTTTGCCTCACTCAGGATTGG–3′
GAPDH	Forward	5′–CACTCACGGCAAATTCAACGGCAC–3′
	Reverse	5′–GACTCCACGACATACTCAGCAC–3′

**Table 2:** Components detected by GCMS analysis of UP.

Retention time	Area (%)	Compound
29.993	20.37	Hexadecanoic acid
51.024	14.83	*β*-Sitosterol
51.861	7.1	2(1H) Naphthalenone
47.525	5.84	1-Naphthalene-sulfonic acid
47.138	3.78	2-Ethylacridine

## Data Availability

The data used to support the findings of this study are included within the article.

## Abbreviations

WAT: White adipose tissue
BAT: Brown adipose tissue
UCP-1: Uncoupling protein 1
PGC-1*α*: Peroxisome proliferator-activated receptor gamma coactivator 1-alpha
LDL: Low-density lipoprotein
HDL: High-density lipoprotein
ALT: Alanine aminotransferase
AST: Aspartate aminotransferase.
